# Ligand Photo-Isomerization Triggers Conformational Changes in iGluR2 Ligand Binding Domain

**DOI:** 10.1371/journal.pone.0092716

**Published:** 2014-04-08

**Authors:** Tino Wolter, Thomas Steinbrecher, Dirk Trauner, Marcus Elstner

**Affiliations:** 1 Department of Theoretical Chemical Biology, Institute for Physical Chemistry, Karlsruhe Institute of Technology, Karlsruhe, Germany; 2 Department of Chemistry, Ludwig-Maximilians-Universität München and Center of Integrated Protein Science, Munich, Germany; University of Bologna & Italian Institute of Technology, Italy

## Abstract

Neurological glutamate receptors bind a variety of artificial ligands, both agonistic and antagonistic, in addition to glutamate. Studying their small molecule binding properties increases our understanding of the central nervous system and a variety of associated pathologies. The large, oligomeric multidomain membrane protein contains a large and flexible ligand binding domains which undergoes large conformational changes upon binding different ligands. A recent application of glutamate receptors is their activation or inhibition via photo-switchable ligands, making them key systems in the emerging field of optochemical genetics. In this work, we present a theoretical study on the binding mode and complex stability of a novel photo-switchable ligand, ATA-3, which reversibly binds to glutamate receptors ligand binding domains (LBDs). We propose two possible binding modes for this ligand based on flexible ligand docking calculations and show one of them to be analogues to the binding mode of a similar ligand, 2-BnTetAMPA. In long MD simulations, it was observed that transitions between both binding poses involve breaking and reforming the T686-E402 protein hydrogen bond. Simulating the ligand photo-isomerization process shows that the two possible configurations of the ligand azo-group have markedly different complex stabilities and equilibrium binding modes. A strong but slow protein response is observed after ligand configuration changes. This provides a microscopic foundation for the observed difference in ligand activity upon light-switching.

## Introduction

Glutamate is the most important excitatory neurotransmitter of the mammalian central nervous system and its receptors play a crucial role in various neural functions [1], [2]. Glutamate receptors fall into two general categories, viz. metabotropic receptors (mGluRs), which underlie slow responses to the neurotransmitter, and the ionotropic receptors (iGluRs), which mediate fast synaptic responses. The iGluRs can be further divided into three subgroups defined by their pharmacology: (i) 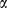-amino-3-hydroxy-5-methyl-4-isoxazole-propionic acid (AMPA), (ii) (2S–3S,4S)-3-(carboxymethyl)-4-prop-1-en-2-ylpyrrolidine-2-carboxylic acid (kainate) and (iii) N-methyl-D-aspartate (NMDA) receptors [3], [4].

The AMPA receptors, which can be composed from four subunits (GluR1–GluR4), are primarily responsible for fast excitatory signaling. After activation of the receptor a nonselective cation channel opens, which is permeable to Na^+^, K^+^ and to small extend also for Ca^2+^.

Most of the structural studies and mechanistic models appeared before a X-ray structure of a functional iGluR2 tetramer in complex with an antagonist was published (pdb code: 3KG2) [5]. These were based on the X-ray structures of the the water-soluble ligand binding domain (LBD) dimers in complex with ligands. A model for the activation of the receptor was proposed, using various ligands, ranging from agonist to antagonists [6]. The comparison of the full receptor structure with the LBD dimer in complex with antagonists shows high similarity, which supports the approach to use the LBD as a model system. The water-soluble LBD dimer is obtained by replacing the first two transmembrane domains, which build the ion channel, by a linker peptide [7] (for illustration see Figure S1 in Supporting Information S1).Thus, the distance of the linker peptides have been considered as a good projection of the ionchannel opening [6].

Ionotropic glutamate receptors (iGluRs) are ligand-gated ion channels that are activated by the excitatory neurotransmitter glutamate. Due to their central role in neuronal signaling, they have been extensively studied and their pharmacology is very well developed. In addition to a large number of agonists and antagonists, many of which are clinically relevant, photo-switchable ligands have recently emerged that allow for the optical control of these important transmembrane proteins.

**Figure 1:**
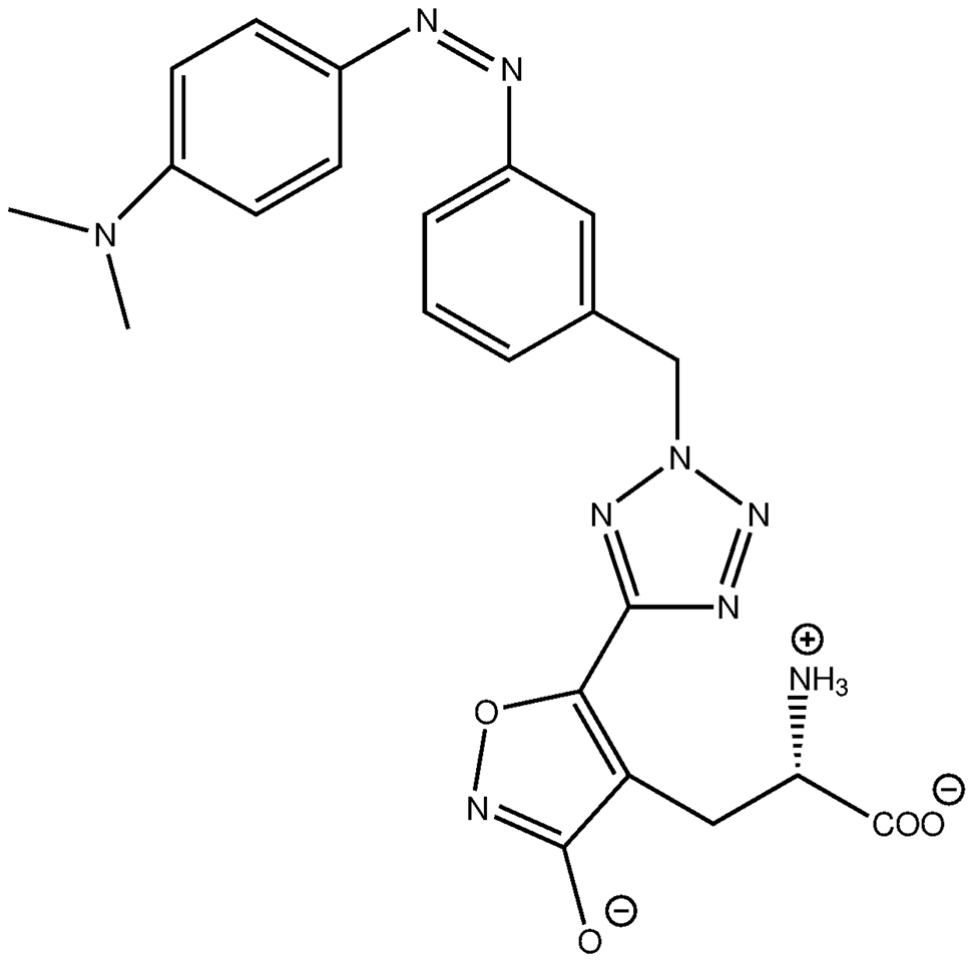
Chemical structure of *cis*-ATA-3.

iGluRs consist of four subunits, each of which contains a clamshell-like ligand-binding domain. Herein, we present a theoretical study on the interaction an iGluR-LBD with a photoswitchable ligand termed ATA-3 (see Fig. 1) [8]. This azobenzene derivative is an agonist in its dark-adapted state (*trans*-form) but looses its activity upon photoisomerization with blue light. However, no structural information or a mechanism is available, which could be used for rational design of these photo-switchable ligands in order to enhance affinity, selectivity or tune the maximum absorption wave length. Up to now, only a schematic picture of the photo-induced process is available, which is based on simple modelling. This provides the idea that the bulky *cis*-conformer does not fit into the binding pocket pocket und therefore moves out the ligand binding domain.

We propose two possible binding modes for this ligand based on flexible ligand docking calculations and show one of them to be analogues to the binding mode of a related non-switchable ligand, 2-BnTetAMPA. In long MD simulations, it was observed that transitions between both binding poses involve breaking and reforming the T686-E402 protein hydrogen bond. We find that the two isomers of ATA-3 have markedly different stabilities and equilibrium binding modes to the LBD of the AMPA receptor GluR2. A protein response is observed after photo-isomerization of the ligand. This provides a microscopic foundation for the observed difference in ligand efficacy upon light-switching.

## Methods

We used Autodock VINA [9] to generate protein-ligand complexes using standard parameters, except the exhaustiveness is increased from 8 to 30. To consider the different possible states of the iGluR2 ligand binding domain (LBD), which range from fully open over several degrees of partial closing to a fully closed state, we used seven different crystal structures [10]. Co-crystallized ligands, if present, range from agonists to antagonists, with pdb codes: 1FTM [6] (AMPA), 2P2A [11] (2-BnTetAMPA), 1FTJ [6] (glutamate), 1FTK [6] (Kainate), 1MQG [12] (IW), 1FTL [6] (DNQX) and 1FTO [6] (APO). According to the available X-ray structure for the whole complex, the LBD is connected to the ion channel via random coils. The distance of the channel to the LBD is about 2 nm and 4 nm to the binding side. Therefore it can be assumed that the ligand binding is independent of the ion transport, i.e. the only function of the LBD is to open the channel, and the opening itself is not influenced by the transported ions. Therefore, we model the LBD opening as an independent process, in accordance to earlier theoretical studies [13]–[16]. Within this study we considered only the LBD monomer, which was done before by other theoretical studies [13]–[16]. Additionally, we tested the introduced error by comparing simulations of monomers and dimers of the LBD with different cofactors. These results suggest that the simplification of a monomer simulation is valid (see Supporting Information S1).

The structural models generated with Autodock were uses as starting structures for MD simulations. All MD simulations were performed with the Gromacs simulation package version 4.5.5 [17]. Protein structures were completed by automatic model building tools, embedded in cubic periodic boxes of 9.7 nm side lengths, solvated with ca. 30,000 TIP3P [18] water molecules and neutralized by adding chloride anions. The Amber99SB force field [19] was used to describe the LBD and ligands were parameterized according to the gaff force field [20] using the Antechamber module of Amber Tools version 11. A time step of 2 fs was used throughout all simulations in conjuction with the SETTLE algorithm to keep covalent hydrogen bonds constrained. A direct space cutoff of 1.0 nm was used for short range van-der-Waals and Coulomb interactions, in conjunction with a PME treatment of long range electrostatics.

All systems built for MD were subjected to an initial equilibration procedure consisting of 500 steps of steepest descent minimization, followed by 500 ps of temperature and volume equilibration to 300 K and average system densities of ca. 1 g/ml. The Nose-Hoover thermostat [21], [22] for heating and the Parrinello-Rahman barostat [23] for equilibration were used throughout. During the 500 ps NVT heating, the protein structure was restrained by harmonic forces of 1000 kJ mol^−1^ nm^−2^. This protocol was carried out to compute 5 trajectories for both possible binding poses (pos. 1 and 2), as detailed below.

**Figure 2:**
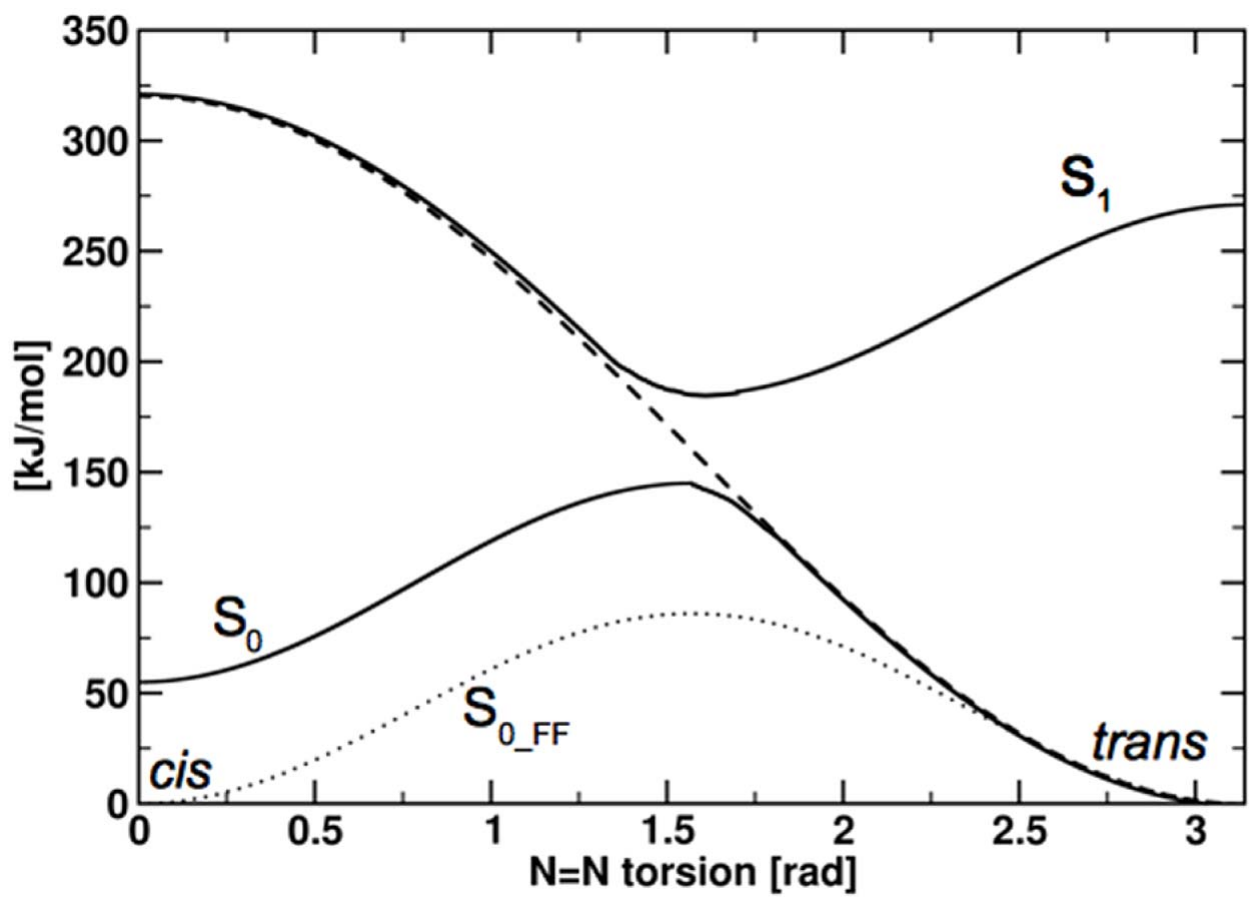
Schematic picture of the potential energy along the torsion around the N = N double bond. The solid lines depict the adiabatic states. The dotted line shows the force field potential and the dashed line represents the switching potential, that mimics the isomerization.

To describe the photo-switching between both azobenzene configurations, we used a simple computational model, since high level *ab initio* methods are very time consuming. The simplified model is applied, since we are interested in the protein response on a long time scale, wile the details of the photo-isomerisation on a short time scale (sub ps timescale) are not the scope of this work. The isomerisation around the N = N double bond is described by a classical potential with 
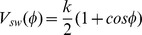
 (k = 320 kJ/mol deg^2^) as suggested by Nguyen *et al.* [24] for similar problems. This potential mimics the energy surface of the S_1_ and S_0_ states along the trans-cis reaction coordinate, as shown in Fig. 2. This switching potential is activated for 500 fs, which is a typical time scale for photoisomerization inside a protein. As the simulations show, this time is sufficient to ‘photoisomerize’ the ligand in all simulations performed so far.

## Results

### Ligand Docking Calculations

Initial protein-ligand complex geometries were obtained from flexible docking calculations. Each ligand was docked using a rigid receptor binding site as well as using two sets of flexible side chains. For the first set, called F1, amino acid residue side chains located close to the natural binding pocket of glutamate in the X-ray crystal structure 1FTJ were selected, namely R405, E402, M708, L650, T686, T655, T480, Y405, Y450. For a second set, called F2, side chains close to the terminal benzyl ring of 2-BnTetAMPA, which is the unswitchable ancestor of ATA-3, in the X-ray crystal structure 2P2A were selected as flexible: I712, Met708, S403, Y405, T686, E402, W767 and Y711. The flexible side chains were chosen by visual inspection with the pre-condition that these could hinder the proper binding due to sterical clashes. Docking calculations were conducted on several X-ray crystal structures of the LBD with different degrees of closing to mimic the induced closure of the LBD, due to ligand binding [10]. A total of seven protein input structures for the LBD of iGluR2 were selected for which high resolution pdb structures were available (see Methods). Each receptor structure was prepared by removing the co-crystallized ligand (if any), assigning protonation states and polar hydrogen positions in Autodock.

We tested the ligand docking approach for this system by re-docking the co-crystallized ligands into the corresponding X-ray structures. In all cases except glutamate, the highest ranked docking pose showed the lowest RMSD compared to the crystal structure. For glutamate, the second highest ranking had the lowest RMSD. RMSD values are typically larger for the systems with a less closed ligand binding domain, presumably due to the higher degree of conformational freedom in the more open structures. A summary of re-docking results is given in Table 1.

**Table 1:** Summary of ligand docking calculations for redocking co-crystallized ligands into their binding pockets.

PDB code	1FTM	1FTJ	2P2A	1FTK	1MQG	1FTL	1FTO
Ligand	AMPA	glutamate	2-BnTetAMPA	kainate	IW	DNQX	none
RMSD [Å]	0.145	0.201	0.329	0.592	0.341	1.045	-
Score [kJ/mol]	−8.1	−6.6	−10.7	−7.8	−8.2	−7.8	-
Rank	1	2	1	1	1	1	

Seven protein X-ray crystal structures (see Methods for references) are listed along with their co-crystallized compounds. ‘RMSD’ gives the root mean square deviation of the best ligand placement compared to the crystal structure position, ‘Score’ the corresponding binding strength prediction and ‘Rank’ the number of the best solution among all docking results for a complex.

**Figure 3:**
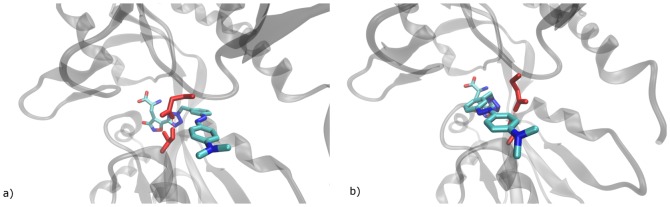
The two proposed binding positions for ATA-3. (a) Position 1; (b) Position 2.

The ligand binding depends on the clam shell closure, which is not know for the novel ligand ATA-3. Therefore, we docked ATA-3 into all of the listed receptor structures, with the photo-switchable azo-group both in its cis and trans configuration (all other rotatable ligand bonds were flexible). For the majority of cases, no reasonable bound geometry was found for ATA-3. Ligand placements on the outside of the protein at the edge of the selected binding site as well as placements forming no hydrogen bond interactions with the receptor, i.e. no hydrogen bonds to the main anchors R485, E705, or to the backbones of S654 and T655 in domain 2, were discarded. For the ligand in its cis configuration, no reasonable docking placements were found at all, while for the ligand in its trans configuration, two possible binding modes were observed. The first, labeled position 1 in the following, was found for the protein X-ray crystal structure 2P2A representing the active state of the receptor with a bound agonist and strongly resembles the binding mode of the 2-BnTetAMPA compound in the crystal structure. The second possible binding mode, position 2, was found when placing trans-ATA-3 into the receptor structure 1FTK, co-crystallized with the partial agonist Kainate. The docking placements position 1 and 2 were found using the F2 set of side chains, while the usage of the rigid protein setup and the F1 set of flexible side chains lead to no reasonable binding structure. A key difference between the two suggested binding modes is their position with respect to the receptor hydrogen bond between T686 and E402. In position 1, the ligand is placed behind this bond while it lies in front of it in position 2 (Fig. 3).

### MD simulations

The ligand docking calculations resulted in two potential binding modes for trans-ATA-3 to the iGluR2 receptor. Based on this, two models of trans-ATA-3 bound to the solvated receptor were built as described above and five independent simulations were conducted for each starting structure. First, models were subjected to the restrained equilibration procedure described in Methods. Equilibration was followed by 500 nanosecond length restraint-free MD-simulations at 300 K for all ten independent simulations.

**Figure 4:**
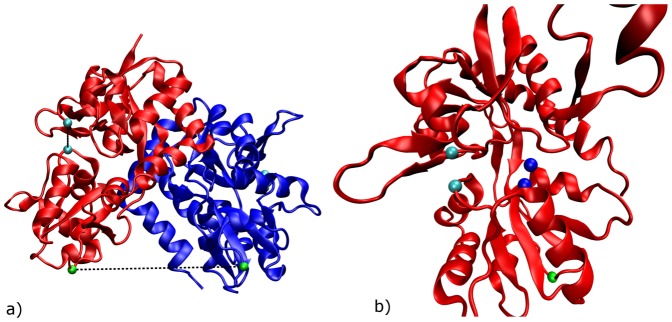
a) LBD dimer with different colored monomers. The cyan spheres depict the center of mass of G451 and S651 within a monomer. The green spheres depict the center of mass of P632. b) LBD monomer. G451 and S651 are depicted by cyan spheres, P632 by a green sphere and the functional groups of E402 and T686 are depicted in blue.

For the ligand in position 1, stable structures were found for both the receptor structure and the position of the bound ligand. RMSD values for the protein uniformly were ca. 0.15 nm after equilibration and rose slightly to ca. 0.2 nm at the end of the simulations. In good agreement with these small RMSD values, no major conformational changes could be observed in any part of the LBD. The protein hydrogen bond between T686 and E402 (Fig. 4b) remained intact during all simulations. Likewise, ligand RMSD values remained at an average of 0.27 nm throughout all five simulation runs and no major changes in the ligand binding mode or its interactions with the binding site could be observed.

For trans-ATA-3 in position 2, greatly reduced complex stabilities were observed: For the initial complex model based on the protein X-ray crystal structure 1FTK (bound partial agonist), the ligand spontaneously dissociated from the binding site after several hundred nanoseconds in all simulations and the ligand binding domain began to open, approaching a structure similar to the apo-protein. To further investigate the possibility of a ligand binding mode similar to position 2, we manually transferred the docked structure of trans-ATA-3 in position 2 of the 1FTK binding site to an analogous position in the binding site based on the protein structure 2P2A. This was done by structurally aligning the receptors based on domain 1, transferring the bound ligand to 2P2A and manually adjusting all side chains exhibiting large van-der-Waals clashes with the ligand. This preliminary complex structure was then subjected to an extended restrained equilibration scheme of 500 ps of MD using 1000 kJ/mol nm^2^ restraints on the backbone of the protein and the non-hydrogen atoms of the ligand, then 10 ns MD with reduced restraints of 100 kJ/mol nm^2^ and 20 ns MD applying restraints of 10 kJ/mol nm^2^.

**Figure 5:**
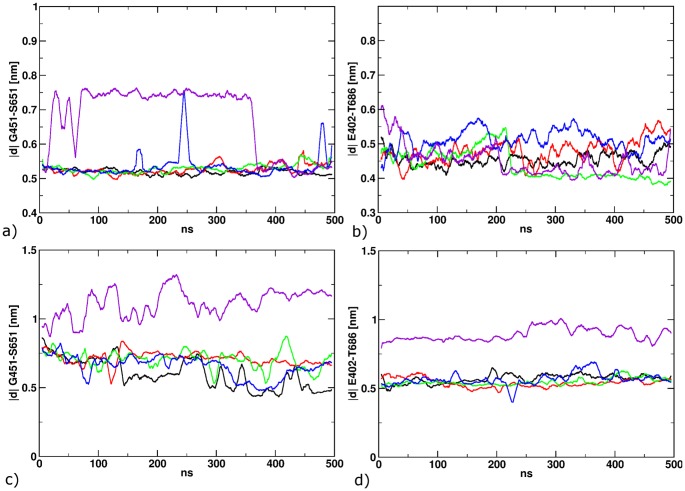
Structural properties along the equilibration MD for POS1 (a+b) and POS2 (c+d). The distance between G451 and S651 describes the opening of the LBD (a+c). The distance between E402 and T686 maps the formation of a hydrogen between the two residues (b+d).

After equilibration, five MD simulations for these newly generated position 2 models were conducted. In four of these, the complex structure remains stable as for position 1 above and for the fifth, partial ligand unbinding was observed (Fig. 5c+d). In the latter case, the protein responds to the ligand unbinding by an increase of the G451-S651 distance to about 1.1 nm, compared to an average of 0.7 nm in the four stable simulations. This distance has been shown to be a good projection of the clamshell motion recently [13] Also, in the fifth simulation, the T686-E402 hydrogen bond opens up, indicated by a distance of ca. 0.8 nm between the center of mass of the functional groups, compared to 0.55 nm for the remaining four trajectories (Fig. 5d).

### Photo-switching Simulations

To simulate the protein response to the *trans-cis* photo-isomerization in ATA-3, we conducted photo-switching simulations using the classical model for the excited state potential energy surface switched on for 500 fs (see Methods). For the ligand in position 1 five MD simulations were performed, using the final structures of the unbiased ground state MD simuations as decribed above. Since in one of the MD simulations for position 2 partial unbinding was observed, only four photoswitching simulations were performed for this case. In all nine simulations, the ligand molecules adopted the cis-configuration during forced switching and maintained it after the external potential was turned off. To study the protein response after ligand photo-isomerization, long unrestrained MD simulations were conducted afterwards.

**Figure 6:**
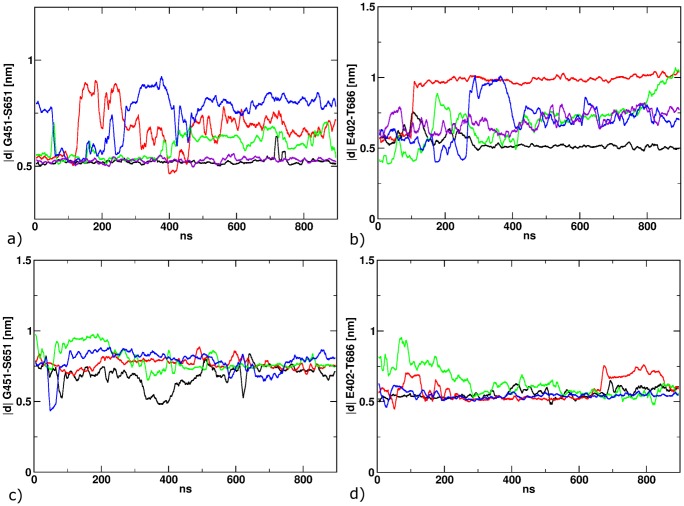
Structural properties after the isomerization to *cis* for POS1 (a+b) and POS2 (c+d). The distance between Gly451 and Ser651 describes the opening of the LBD (a+c). The distance between E402 and T686 maps the formation of a hydrogen between the two residues (b+d).

For the four simulations starting in position 2, switching the ligand into the cis-configuration does not generate a notable protein response. Both, protein backbone and ligand binding mode, remain stable over 900 ns of simulation time relative to the starting structure. Likewise, the T686-E402 hydrogen bond (see Fig. 6d) remains closed at an average distance of 0.6 nm.

**Figure 7:**
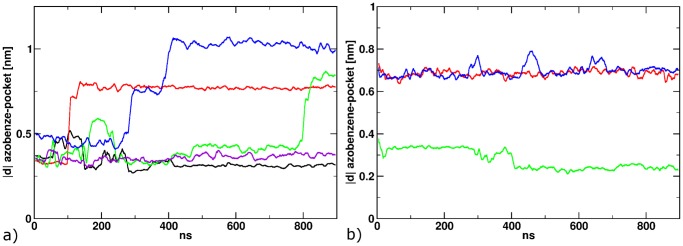
The distance between the azobenzene and the binding pocket, that describes the translocation from position 1 to position 2. a) 900 ns after the isomerization to *cis*; b) 900 ns after back-isomerization to *trans*.

In the five simulations after switching the ligand starting in position 1, a slow but marked protein response and ligand conformational rearrangement is found (Fig. 6a+b). The distance between G451 and S651 increases in three of five cases, however, to a different amount (Fig. 6a) Large conformational rearrangements are also found for the ligand molecules. The cis-ATA-3 ligand remains in the position 1 binding mode in one case (Black curve in Fig. 6a+b). In a different simulation (purple curve) the ligand stays in position 1, but interrupts the T686-402 hydrogen bond due to rotation of the *cis*-azobenzene (Fig. 6). In three cases (red, green, blue) the azobenzene passes the T686-E402 hydrogen bond after 100, 300 and 800 ns simulation time and move towards position 2. To monitor this motion, we projected the path from position 1 to position 2 onto the distance between the center of mass of the azobenzene and the pocket, defined by residues S403, P404, Y405, T707, Y711 and I712 (Fig. 7a). Comparing this distance and the breaking of the hydrogen bond between T686-E402 one can observe a clear dependency. Furthermore, the azobenzene/pocket distance depicts, that only in one of the three simulations position 2 is reached at ca. 1.05 nm blue curve). The other two simulation end in an intermediate state at around 0.75 nm.

**Figure 8:**
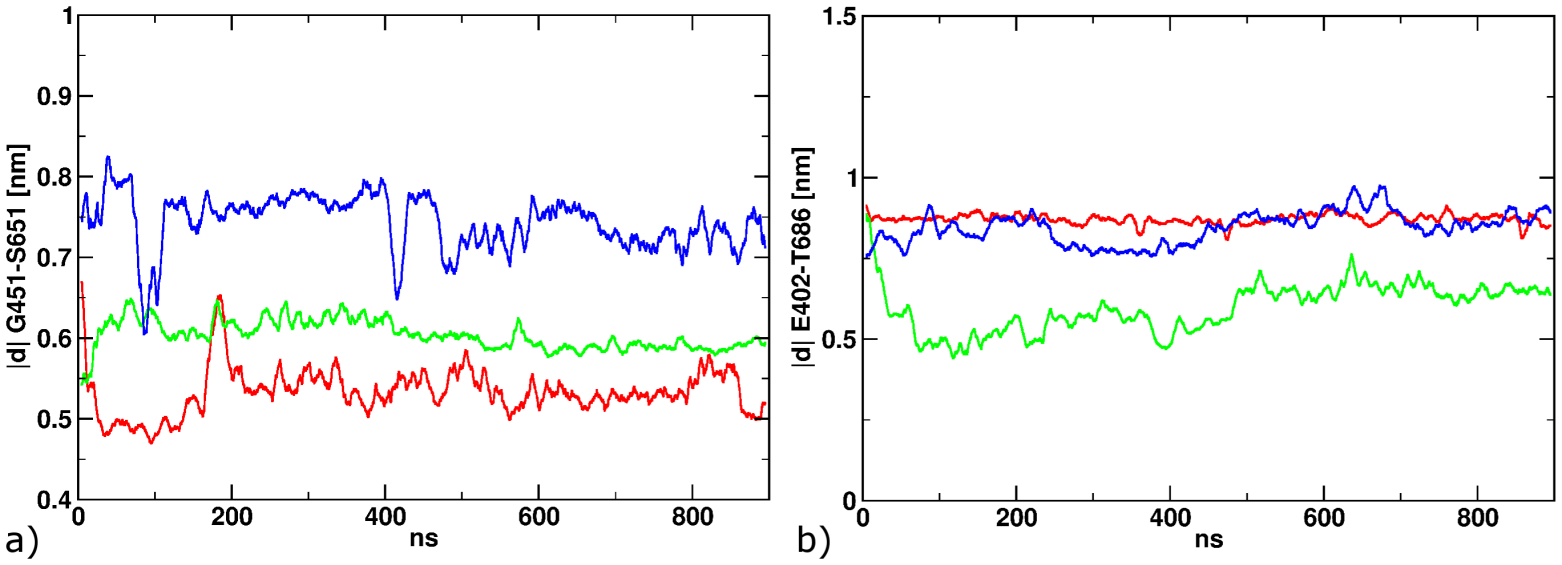
a) The distance between Gly451 and Ser651 after the back-isomerization to *trans*; b) The distance between E402 and T686 after back-isomerization.

Since the experiment [8] implies a reversible mechanism, we simulated the reversed process for the three cases in which the ligand changed its binding mode from position 1 to position 2. Starting from the final structure of the 900 ns length simulations with the cis-ligand, we again applied a harmonic dihedral angle biasing potential for 500 fs, this time with the potential minimum corresponding to the trans-configuration. In one of the three cases, a direct reorientation into position 1 (Fig. 7b; green curve), accompanied by the formation of the T686-E402 hydrogen bond (Fig. 8b) can be observed. In the two other simulations, only an intermediate position (red and blue curves) is reached, where the ligand is halfway between the positions 1 and 2, being located between the residues E402 and T686, thereby preventing the formation of a hydrogen bond between these residues. This probably is an intermediate, for longer simulations we would expect the transition to position 1.

The photo-switching simulations show, that ligand binding position 1 is stable for the ligand in trans-configuration only and that it spontaneously changes towards binding position 2 upon isomerization. This conformational change is reversible on the microsecond time scale in unbiased MD simulations, but no quantitative information about the potential energy landscape of the complex can be obtained from the limited number of simulations.

We have therefore conducted free energy calculations on the conformational changes between the ligand positions (Supporting Information S1). This leads to massive convergence and hysteresis problems, which is shown in the supplementary materials section. This might be caused by one dimensional reaction coordinate, which describes only the ligand reorientation but not the opening/closing of the LBD. The usage of a 2-D Umbrella Sampling might help to overcome this problem. However, this would lead to over 300 windows, which is computationally too demanding in the moment, while a better convergence is not guaranteed.

### Reaction of the ion channel

All simulations were performed only for one monomer. For further analysis, we projected the second monomer onto the monomer trajectories in such a way that the two domains 1 have the same alignment like in the crystal structure and measured the distance between the linker residues P632, that are inserted into the LBD to replace the transmembrane sequence of the receptor (Fig. 4) This distance is a good measure for the opening of the ion channel. For MD simulations of the crystallized LBD complexes, we observed a high correlation (R^2^ = 0.916) between the G451-S651 and the P632 distances, i.e. clam shell motion and ion channel opening are highly correlated.

**Figure 9:**
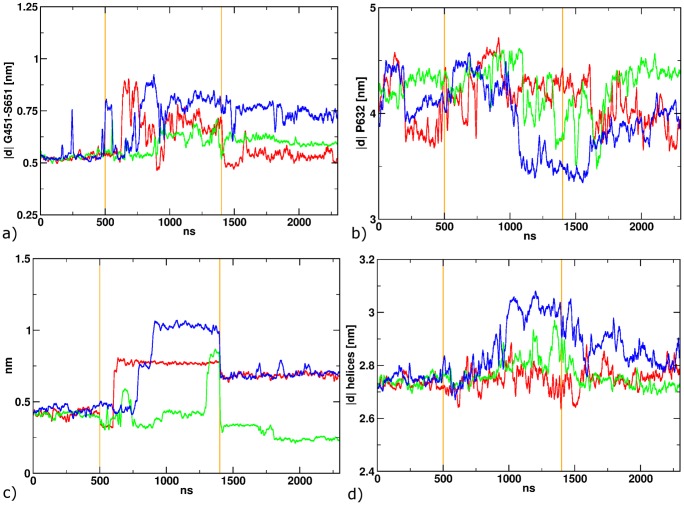
Different structural properties along the three consecutive MDs. The vertical orange lines depict the isomerization. a) G451-S651 distance; b) P632 distance; c) Distance between the azobenzene and the pocket; d) distance between helix H and helix B.

**Figure 10:**
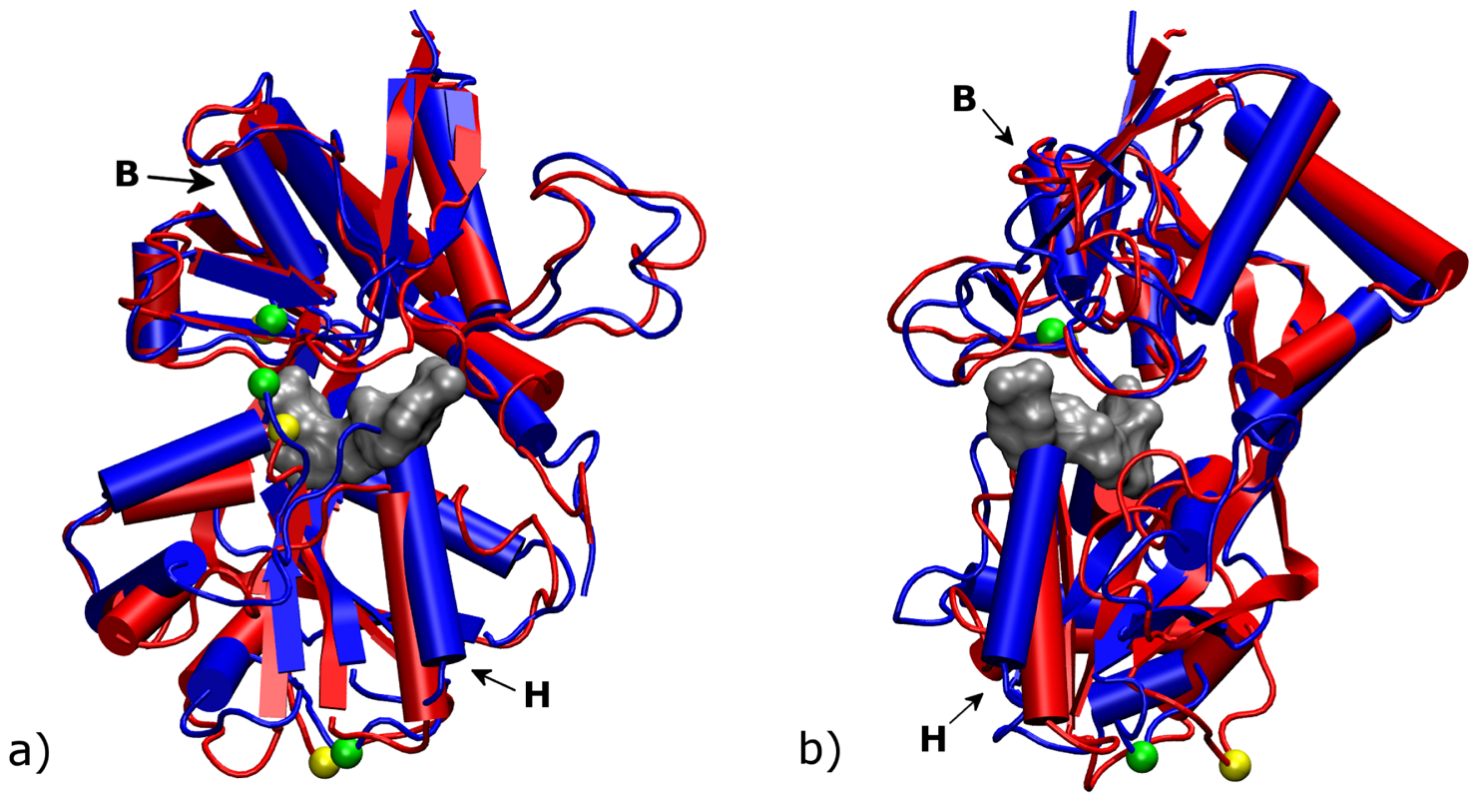
Illustration of the push down of helix H. The blue protein structure is taken after equilibration run. The red protein structure is caused by *cis*-ATA-3 (grey bulk) in position 2. a) Front view; b) Side view.

In contrast, for our three productive photo-switching MD simulations, we do not find a significant correlation (R^2^ = 0.11) of clam shell motion and channel opening (Fig. 9a+b). However, comparing the distance between the azobenzene and the pocket (Fig. 9c) and the linker distance (Fig. 9b) a correlation can be found. When the ligand moves from position 1 towards position 2, the distance between the linkers decreases with a delay of ca. 100 ns and vice versa. An alignment of a starting protein structure with a ligand in position 1 and one with the ligand in position 2, depict the effect of the ligand relocation (Fig. 10). Due to the relocation of the azobenzene, helix H is pushed down, which decreases the distance between the linkers. We monitored this push down along the trajectories by measuring the distance between the centre of mass of helix H and B, which shows a similar shape like the azobenzene-pocket distance.

## Discussion

### 1.1 Ligand Docking Calculations

Autodock Ligand Docking Calculations with a partially flexible binding site selection were able to generate chemically reasonable protein ligand complex geometries for ATA-3 binding to the iGluR binding domain. The reliability of ligand docking results was validated by re-docking a series of seven ligands of known structure and obtaining excellent agreement of the top ranked placement to the known binding geometry. The single exception, the natural ligand glutamate for which only the second placement corresponded to the experimental binding mode, can be understood as well, since the glutamate ion is a small, highly polar molecule very unlike typical drug compounds for which docking tools are developed and may therefore represent a case were the autodock scoring function is unreliable. Even in this case, the correct binding mode is found, if not ranked perfectly. Using a variety of experimentally known receptor structures corresponding to different levels of LBD closure gives quite different docking results for ATA-3. No binding poses were suggested for the cis-configuration of the ligand. Only for one receptor X-ray structure (2P2A) we found a binding mode of trans-ATA-3 that resembles that of the similar compound 2-BnTetAMPA. For one additional, slightly more open receptor structure (1FTK), a second, unexpected binding pose was predicted for trans-ATA-3. This suggests that i) we have found a credible binding mode and a second, possible metastable, binding mode (position 2) for trans-ATA-3 and ii) the cis form of the photo-switchable ligand is difficult to place into the LBD, a possible source for its change in activity. Both points indicate that receptor-ligand complex dynamics play an important role here. We have therefore proceeded in conducting all-atom molecular dynamics simulations of the systems.

### 1.2 MD Simulations

500 ns long MD simulations show that both suggested ligand binding poses lead to stable solvated complex structures. ATA-3 bound in position 1 was observed to form a complex that exhibited no significant conformational changes in five independent simulations. LBD complexes of ATA-3 bound in position 2 exhibited higher degrees of flexibility. In the initial starting structure, ligands spontaneously unbind from the LBD over hundreds of nanoseconds in several independent simulations. A second complex model of ATA-3 in position 2, based on the closed LBD structure 2P2A, showed stable complex binding modes over 500 ns length, but still one out of five independent simulations showed the ligand spontaneously dissociating from the LBD. This agrees with the hypothesis that binding position 2 corresponds to a metastable bound pose, unlike the tight binding in position 1.

### 1.3 Photo-switching

The photo-switching simulations adopted a protocol used previously on this system, where a short biasing potential simulates the ligand photo-reaction and the following slow protein response can then be monitored in MD simulations. In principle, the full photo-reaction could be simulated using appropriate QM/MM models, but due to the large time scale separation between photo-switching (on the fs timescale) and receptor response (involving many ns to 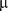
s of dynamics), a classical model can be used to study receptor conformational changes. We find that switching the ligand in binding position 2 is not generating a noticeably response of the LBD. this further suggests that position 2 is a transient binding mode lacking strong ligand-receptor fit and interactions. In contrast, switching ATA-3 to its cis form in binding position 1 does generate a significant complex response. Interestingly, we find that trans-cis isomerization forces the ligand to adopt binding position 2 in several simulations. This process appears to take on average many hundreds of nanoseconds, since it is only observed in three out of five simulations after 900 nanoseconds. It is not clear if binding position 2 represents a stable binding mode of cis-ATA-3 or if we only observe the initial stages of complete ligand unbinding. The lack of ligand docking poses for cis-ATA-3 suggests the later. The slow protein reaction due to the photoswitch shows that the possible unbinding of the *cis*-conformer is not caused by sterical clashes, e.g. mechanical force. Even the bulky *cis*-azobenzene fits into the binding pocket without causing a fast clamshell opening or dissociation of the ligand. Thus, we could show that simple mechanical models are not appropriate, which is surprising at first glance since the geometry between *trans* and *cis* seems to quite large. However, the isomerization of the chromophore does not lead to immediate changes in the binding pocket. Such a behavior is well know from other photoreceptors like rhodpsins. A surprising observation is the complete reversibility of the position 1→2 binding mode change. Additionally, the function of the T686-E402 hydrogen bond acting as a gate between both conformations can be clearly seen from the repeated opening and closing of the interaction when ligand pass between binding positions.

### 1.4 Reaction of the ion channel

The comparison of several structural parameters could show, that the first changes in the binding pocket accompanied with the photo-isomerization, do not trigger the clamshell motion of the LBD. We could present a spatially limited protein reaction, which includes mainly one helix. However, the motion of this single helix already has an impact on the ion channel. It has to be mentioned, that despite the long simulation time, subsequent processes like ligand unbinding from position 2 and the classical LBD opening, might be not obtained, because of the lack of statistics. Moreover, the simulation of one LBD monomer could lead to artefacts, because of the neglected interactions with the ion channel and the other monomers.

## Conclusion

Computational modeling allows a detailed description of processes following the photo-switch reaction of iGluR2 bound ATA-3 ligands. We suggest that a stable ligand binding mode exists, similar to that of other known ligands of similar composition. In addition, a second less stable binding mode exists, differentiated from the global minimum by the ligand passing through a crucial protein hydrogen bond, which is adopted by the ligand immediately after a change to its *cis* form. This second binding mode often results in complete ligand dissociation from the LBD, but the position change is fully reversible if the ligand changes back into its trans form after less that a microsecond. In the second, metastable binding pose, the ligand does not have significant effects on the LBD conformation anymore. Movement of a photo-switchable ligand between the two positions, followed by possible complete unbinding, is therefore a good explanation of the different activities of cis and trans forms of ATA-3 and can serve as a microscopic model for the photo-switch effect.

## Supporting Information

Supporting Information S1 Within the Supporting Information S1 we present additional simulations of the LBD dimer, which support our simplification of the system. Furthermore, the results and technical details of our free energy calculations are shown.
(PDF)
