# Supplementary material for: Ligand Photo-Isomerization Triggers Conformational Changes in iGluR2 Ligand Binding Domain
Source: PLoS One. 2014 Apr 8;9(4):e92716. doi: 10.1371/journal.pone.0092716 (PMC3979659; doi:10.1371/journal.pone.0092716)
Supplement: Supporting Information S1 — Within the Supporting Information S1 we present additional simulations of the LBD dimer, which support our simplification of the system. Furthermore, the results and technical details of our free energy calculations are shown. (PDF) [file pone.0092716.s001.pdf]

Ligand Photo-Isomerization Triggers Conformational Changes in iGluR2 Ligand Binding Domain - Supporting Materials

Tino Wolter[1], Thomas Steinbrecher[1], Dirk Trauner[2], Marcus Elstner[1,*]

1 Department of Theoretical Chemical Biology, Institute for Physical Chemistry, Karlsruhe Institute of Technology, Kaiserstr. 12, 76131 Karlsruhe, Germany

2 Department of Chemistry, Ludwig-Maximilians-Universität München and Center of Integrated Protein Science, Butenandtstr. 5-13, 81377 Munich, Germany

∗ **E-mail: marcus.elstner@kit.edu**

Introduction-Supporting Materials

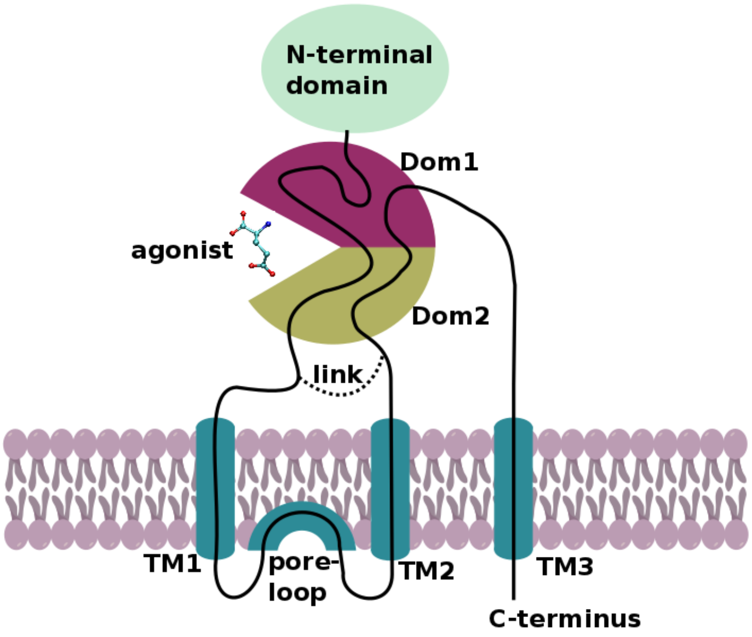

Figure S1. Schematic representation of an iGluR monomer

Dimer Simulations

To analyse the error introduced by simulating only the monomer, we performed long time MD simulation of the LBD co-crystallized with known the known ligands AMPA, 2-BnTetAMPA, DNQX and the apo state in their monomer and dimer form. The monomers were simulated for 1 μs while the simulation time of the dimers was reduced to 300 ns due to computational time.

The RMSD between the monomer and dimer simulation have been calculated (see Table S1). The small RMSD values for AMPA, BnTetAMPA and DNQX shows that the overall structure does not suffer from simulating only a monomer. In case of the APO form, the high RMSD can be explained by the huge LBD opening during the monomer simulation, which we already described in ref. [13]. This huge clamshell opening cannot be observed during dimer simulation, because this would lead to sterical clashes. A more severe difference is the reduced correlation between the internal motions within the monomers and the Pro632 distance . The explanation for this reduced dependency is not structural changes within the single monomers, but in the motion of the monomers to each other. A PCA of the dimer dynamics depict two mayor principal modes, which cover already over 80 % of all fluctuations. The corresponding motions are located only between the monomers and not within. The first principal mode describes a shearing motion. The second mode describes the motion of a clothespin. A schematic picture of both motion are shown in Fig. S2. Thus, an analyze of the correlation between the interdomain motions within a LBD monomer and the channel opening is hard to achieve. Furthermore, it is disputable if these motions can be observed in the native receptor. First, the LBDs are connected to the ion channel and a huge N-terminal domain, which should reduce these motions. Second, the 4 monomers are not arranged in parallel, but are interlaced, which further suppresses these motions. Since the introduced errors by simulating a monomer are limited to the wide opened form of the LBD, we are confident that our monomer simplification is valid in the range of normal LBD opening.

Table S1. RMSD between the monomer and dimer simulation. For calculation of the RMSD the end structures of the MD simulation have been used. All values in nm

	Dimer chain A	Dimer chain B
AMPA monomer	0.19	0.18
BnTetAMPA monomer	0.08	0.10
DNQX monomer	0.19	0.17
APO monomer	0.39	0.35

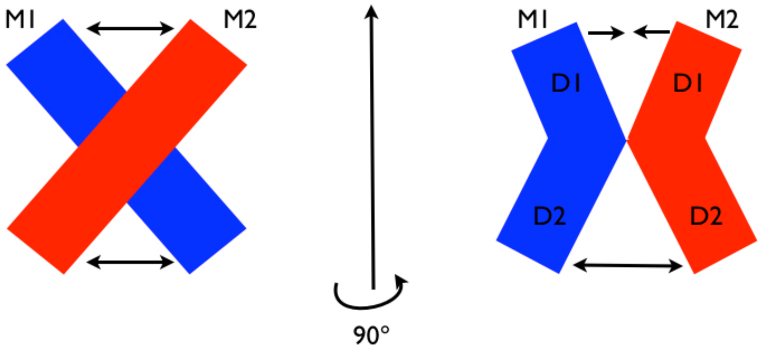

Figure S2. A schematic representation of the first two principal motions of the LBD dimers. Left: The shearing motion; Right: clothespin motion.

Umbrella Sampling

Umbrella Sampling Calculations

Umbrella Sampling (US) calculations were set up to determine potential of mean force curves for ligand movement between binding positions 1 and 2. A one-dimensional reaction coordinate was used, in which binding position 1 corresponds to a distance of 0.5 nm and position 2 to a distance of 1.1 nm. It is defined by the distance between the center of mass of the azobenzene and the pocket (defined by residues S403, P404, Y405, T707, Y711 and I712). This reaction coordinate was sampled using 21 equally spaced umbrella windows, each with a harmonic restraining potential of 2000 kJ/mol nm^2. Starting structures for all windows were obtained from 100 ns long simulation, with a pull rate of 10^{-6} nm/ps. Three different US simulations were set up: One for moving the ligand in its cis-configuration from position 1 to position 2, one for the reverse change, moving the cis-ligand from position 2 to position 1 to test for hysteresis effects. A third simulation moving trans-ATA-3 from binding position 1 to position 2. US simulations were conducted as consecutive series of 100 ns length simulations for each independent window. Simulation convergence was judged by checking histogram overlap along the reaction coordinate as well as monitoring the change in the resulting PMF curve after each additional 100 ns of data was included.

In Figure S3, the most favorable binding position for the trans-ligand is clearly position 1, deep in the receptor cleft, behind the crucial E402-T686 hydrogen bond. A high energy barrier to remove the trans-ligand from binding position 1 is observed. Several local minima are observed when moving the

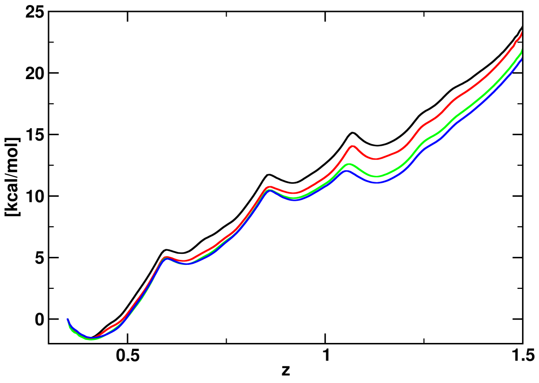

Figure S3. Free energy profile for the transition of *trans*-ATA-3 from POS1 to POS2

ligand to binding position 2 but overall, position 1 is found to be the preferred binding mode by more than 12.8 kcal/mol.

The PMF for the same transition with *cis*-ATA-3 conflicts with our observation during the free MD simulations (Fig. S4). Between 0.3 nm and 0.75 nm, one can see a very broad energy plateau with two minima at 0.375 and 0.55 nm, which are separated by a barrier of 0.75 kcal/mol. These minima correspond to position 1 and an intermediate, where the azobenzene is located between E402 and T686. This broad energy plateau leads to an saddle point at 0.93 nm (+7.1 kcal/mol) and a minimum at 1.07 (+8.3 kcal/mol) that resembles position 2. This strong increase of the free energy accompanied with the transition to POS2 stands in contrast to the free MD simulations, which suggest a lower free energy for position 2 than for position 1. Moreover, the PMF is not converged within the 300 ns. In the region

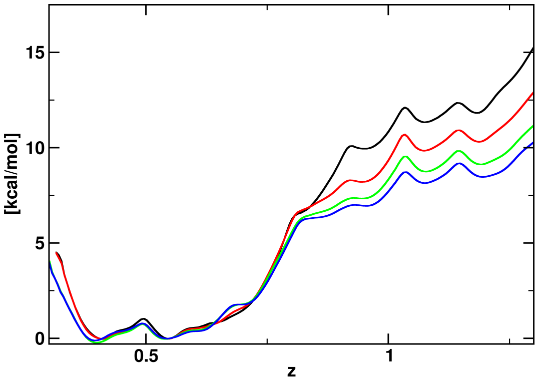

Figure S4. Free energy profile for the transition of *trans*-ATA-3 from POS1 to POS2

around 1 nm the energies changed about 0.8 kcal/mol within the last 50 ns. It is known, that the Umbrella Sampling method suffers from the occurrence of hysteresis, e.g. the free energy profile differs between

an A-to-B and a B-to-A transition. To estimate the effect of hysteresis for this reaction coordinate, we performed an Umbrella Sampling with the same reaction coordinate, but starting from position 2 and pulling towards position 1.

The resulting PMF, depict a strong hysteresis for this reaction coordinate. Position 2 is energetically favored over position 1. Moreover, the PMF has no minimum around 0.375 nm, but a steep increase in free energy. This might be produced by the fact that the hydrogen bond between E402 and T686, which is normally present in POS1, is not formed spontaneously during the US simulations. In general, the PMF is very flat and could not explain the long simulation times, which are needed to monitor the transition from POS1 to POS2. The contradicting PMFs raise the question, if a one-dimensional reaction

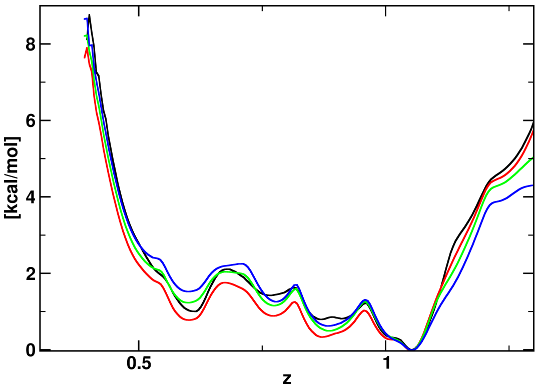

Figure S5. Free energy profile for the transition of *trans*-ATA-3 from POS2 to POS1

coordinate, like we have selected, is capable to describe the energetics of such a transition.

Umbrella Sampling Discussion

Umbrella Sampling Free Energy calculations were used to obtain PMF curves for the ligand binding position changes. Despite extensive simulation lengths, considerable hysteresis effects were observed, even though individual US transformations appear converged. Presumably, even microsecond length MD simulations are insufficient to fully converge free energy calculations starting from different receptor structures. Therefore, the resulting free energy curves should be taken as qualitative information only.

Moving the trans form of the ligand between binding positions 1 and 2 clearly shows that position 1 is the more stable global free energy minimum conformation, separated by a high barrier (corresponding to the breaking of the T686-E402 hydrogen bond) from the next lowest minimum, position 2. This again suggests that ATA-3 adopts a binding mode very similar to that of the comparable 2-BnTetAMPA

ligand. In contrast, simulations of the ligand in its cis form show a significantly lowered energy barrier to change binding positions, in good agreement to the observed spontaneous changes in binding mode. The simulations of cis-ATA-3 show a smaller free energy difference between binding positions 1 and 2, but it is unclear which would be the preferred binding mode (if any) of the cis form.
